# Philadelphia County Medical Society

**Published:** 1873-04

**Authors:** 


### PHILADELPHIA COUNTY MEDICAL SOCIETY.

At a conversational meeting held November 13, 1872, at eight o'clock P.M.—Dr. J. G. Stetler, Vice-President, in the Chair—Dr. Washington L. Atlee reported the following case: On the 23d of last September he was called to see a lady, sixty-three years of age, who came to the city from the interior of the State. Previously she never had any disease, and she had no hereditary taint or morbid diathesis of any kind. During the past winter she took cold easily, and complained of general *malaise.* Early in March last, the left side of her throat became affected, the submaxillary gland on the left side enlarged. Tumors then formed in both axillæ, in the parotid regions, and in the groins.

When he saw her first, on the day above mentioned, she was in a cachectic condition, could not articulate, and deglutition and breathing were difficult. The tumor on the left side extended from the external ear to the shoulder; on the right side, the tumor was only one-half this size. The sublingual and submaxillary regions were both enlarged. The axillæ were so filled up that the arms stood out from the body a considerable distance. The upper and lower extremities were œdematous. The mouth was filled with a tumor from its middle back into the pharynx, thrusting forward the soft palate, so as to make it become the anterior and upper wall of the tumor, interfering with respiration, deglutition, and speech.

She came to me, thinking to have it extirpated. On the surface projecting beneath the soft palate, there was an ulceration the size of a silver half-dollar. These tumors were so hard and immovable that, had it not been for their general distribution, Dr. A. would have considered the disease to be scirrhous cancer. An isolated tumor of such a character, occurring at this age, and accompanied by such decided cachexia, he would not hesitate to pronounce malignant. The growth had been exceedingly rapid, and different modes of treatment, including electrolysis, had been tried, without any improvement. Hesitating to call it malignant, he had given an unsatisfactory diagnosis and an unfavorable prognosis; but to-day the patient is well, the tumors having disappeared almost entirely. The treatment had been simply three drops of Fowler's solution and ten grains of the muriate of ammonia three times a day, with a locally-applied solution of muriate of ammonia.

Dr. Atkinson spoke of a case in the person of a woman with a child six months old. He had first seen her three months ago, and had been attending her at the Howard Hospital. He considered the trouble to be an ovarian tumor, and had been prescribing ten grains of muriate of ammonia three times a day. On this statement of Dr. Atlee, he would continue it, with the addition of three drops of Fowler's solution three times a day.

Dr. O'Hara asked if these swellings were not adenoid enlargements from chronic adenitis? He recalled a case in which there were persistent tumors on both sides of the neck, resembling, on a small scale, Dr. Atlee's case, in which arsenic, iron and iodide of potassium had been used for months, but which now seems to be improving under iodide of mercury and cod-liver oil.

He also mentioned, in connection with the subject of arsenic, that, owing perhaps to idiosyncrasy, arsenic at times, like mercury, can be exhibited and continued in almost enormous amounts. One of his patients has taken arsenic for twenty years, at intervals, at his own discretion, for psoriasis. To his knowledge, he has taken one-tenth of a grain three times a day for six months, always with benefit to the skin affection and with no apparent ill effects. He asked whether any gentleman had used decolorized tincture of iodine externally? By the addition of hyposulphites to the tincture, it can be made transparent, and its external application would not stain the skin. He also added that he had seen a case of epithelial cancer of the vagina, pronounced such by himself and an eminent professor, which seemed to be cured by arsenic and the local application of iodine and the permanganates. This patient subsequently bore a child, and locally there is no trace of the disease.

Dr. Atlee said that his case was neither one of adenitis nor of parotitis, as the tumors were not sensitive, and the patient complained of no pain. Neither was it scrofulous, as scrofula could not be first developed at such an advanced age, nor would it yield so readily to treatment. Neither could he regard the disease as adenoma. He did not know what it was, and desired to be informed by some of the experienced gentlemen present. He would have called the disease scirrhous cancer, had it not been so generally distributed through the system. The contents of the tumor were not microscopically examined. He believed that the cancer-cell could be kept from forming by using arsenic in small doses, and keeping the patient under its tonic and alterative action, avoiding its specific influence on the system, as well as gastric irritation, and continuing it for a long time—one, two or three years.

Dr. W. M. Welch asked whether it was not possible for this tumor to have been an enlarged lymphatic gland, deeply seated, pressing forward internally?

Dr. Atlee said the tumors were entirely distinct and somewhat nodulated.

He also stated that her sister, who is two or three years older, has goitre. For goitre, he always uses the officinal tincture of iodine, ten drops three times a day, together with its external application. He has never found this to fail, but has had to continue the treatment one, two or three years. With regard to arsenic, he wished to be distinctly understood; he does not believe that it will cure cancer. If, however, the cancerous growth be removed, which he believes in many cases to be a local disease, then it is that arsenic will show its remedial power. It will extinguish, he believes, the tendency to cancerous formation in that part or in any other. It will not produce absorption of cancerous material, but simply prevents its development. In several instances of cancer of the breast, after amputation, he has seen, under this treatment, the patients remaining well from five to twenty-five years. In one case of carcinoma uteri which had been pronounced incurable by Professors Meigs and Gilbert, he had applied to the diseased surface every four days the following—℞. Iodinii, ʒj.; potass. iodidi, ʒj.; glycerinæ, fʒij.—while internally arsenic was administered. This patient was treated several years ago, and is now well. She is over fifty years of age. Similar cases have been treated in their incipient stage with like results. He referred to another case—that of the wife of a physician of this city—in whom the cancerous growth involved the lower portion of the cervix and os, the vaginal fold surrounding the neck not being invaded. She was in a cachectic condition, with anæmia from repeated hemorrhage. The diseased cervix was removed several months ago, and the arsenical treatment adopted. The parts are now in a healthy condition, and the patient goes out daily. The arsenical treatment will be continued for a year or two. If there is no deposit on the vaginal wall, he would recommend the removal of the os and cervix, even though there was a possibility of some diseased tissue remaining above the point amputated. The patient will recover from the operation, her life be prolonged, and her health much improved; but the disease will again return at some future time.

Dr. W. H. Pancoast stated that he had witnessed the use of condurango in the case of a gentleman affected with an encephaloid cancer of his upper jaw. He was called in too late to perform any operation for the removal of the diseased part, and could only recommend cleansing applications, together with a supporting and soothing treatment. The patient finally died of exhaustion. He had been taking, upon his own responsibility, without any benefit, a wineglassful of the fluid extract of condurango three times a day for more than six months.—*Philadelphia Medical Times.*

